# Diagnostic and Therapeutic Challenges Between Peripartum and Influenza-Induced Inflammatory Cardiomyopathy—A Case Report and Literature Review

**DOI:** 10.3390/jcm14103440

**Published:** 2025-05-14

**Authors:** Karolina Stachyra, Monika Zasztowt-Sternicka, Magdalena Litwinska, Ewelina Litwinska-Korcz, Izabela Walasik-Szewczyk, Zoulikha Jabiry-Zieniewicz, Monika Szpotanska-Sikorska

**Affiliations:** 1st Department of Obstetrics and Gynecology, Medical University of Warsaw, pl. Starynkiewicza 1/3, 02-015 Warsaw, Poland

**Keywords:** peripartum cardiomyopathy, inflammatory cardiomyopathy, influenza

## Abstract

**Objectives:** Peripartum cardiomyopathy (PPCM) is a life-threatening cause of heart failure in late pregnancy or postpartum, often difficult to distinguish from other types of cardiomyopathies, such as influenza-induced inflammatory cardiomyopathy (ICM). This case report highlights the diagnostic challenges of differentiating PPCM from ICM and the management of this condition. **Methods:** A retrospective case analysis was conducted based on medical records from a tertiary centre in Warsaw, Poland, with a follow-up via phone consultations. A literature review was performed using PubMed, Scopus, and Google Scholar, incorporating relevant European Society of Cardiology guidelines. **Results:** A 34-year-old woman with a twin pregnancy at 36 + 5 weeks underwent a caesarean section and later experienced a syncopal episode. Elevated cardiac biomarkers and inflammatory markers suggested myocardial injury. Echocardiography showed reduced left ventricular function, raising concerns for PPCM or ICM. Cardiac magnetic resonance revealed left ventricular dysfunction without myocardial inflammation, supporting a PPCM diagnosis. Despite LVEF recovery to 65%, a Holter ECG at seven months postpartum showed persistent arrhythmias, necessitating referral for ablation. **Conclusions:** This case emphasizes the need for a thorough diagnostic approach to differentiate PPCM from conditions like ICM. Long-term monitoring, pre-conception counselling, and preventive strategies, such as influenza vaccination, are crucial for managing PPCM and preventing future complications.

## 1. Background

Cardiovascular conditions, including heart failure, are among the leading non-obstetric causes of maternal mortality, with approximately 60% of heart failure cases occurring postpartum [1]. The differential diagnosis of heart failure during pregnancy includes conditions such as pre-eclampsia, myocarditis, Takotsubo cardiomyopathy, pre-existing or familial cardiomyopathies, and pulmonary embolism. However, peripartum cardiomyopathy (PPCM) is indeed one of the most common causes of heart failure in pregnant women [2]. These conditions must be carefully evaluated through patient history, imaging, and laboratory findings in the diagnostic process [3].

PPCM is an uncommon, estimated to be 1 per 1500–5000 deliveries in Caucasians, yet serious form of heart failure that manifests in the final month of pregnancy and within months following childbirth. It is characterized by left ventricular systolic dysfunction with no identifiable cause [4]. On the other hand, inflammatory cardiomyopathy (ICM) is a condition where myocardial inflammation leads to cardiac dysfunction. This inflammation can be caused by various factors, including infections, autoimmune diseases, and exposure to certain toxins [5]. Influenza-related myocarditis is considered as a cause of inflammatory cardiomyopathy, particularly in the context of influenza A infection [6]. The virus can directly infect myocardial cells, induce an excessive inflammatory response leading to damage of the heart muscle or cause ventricular dysfunction resulting from reduced oxygen levels due to respiratory infection [7,8].

## 2. Objectives

- To discuss the differential diagnosis of peripartum cardiomyopathy and inflammatory cardiomyopathy, highlighting key diagnostic tools.
- To describe a clinical case of peripartum cardiomyopathy and the challenges in diagnosing and managing the condition.
- To emphasize the importance of long-term monitoring and preventive strategies for PPCM patients.

## 3. Materials and Methods

The case report was based on the patient’s medical records retrieved from the clinic’s medical system, which included clinical assessments, diagnostic tests, and treatment interventions. The follow-up was conducted via phone calls to monitor the patient’s progress and gather additional information regarding her ongoing condition.

The literature review supporting the case was conducted using PubMed, Scopus, and Google Scholar with the search terms “peripartum cardiomyopathy”, “inflammatory cardiomyopathy”, “influenza cardiomyopathy”, “influenza myocarditis”, and “pregnancy heart failure”. In addition, relevant guidelines were consulted, including the “Guidelines for the Management of Cardiovascular Diseases During Pregnancy” presented by the European Society of Cardiology (ESC) [9,10].

## 4. Case Presentation

A 34-year-old woman in her second pregnancy, having previously delivered one child without any complications, with a twin monochorionic diamniotic pregnancy at 36 + 5 weeks of gestation, was admitted to the hospital for a planned caesarean section due to non-obstetric reasons. Her medical history included two recent upper respiratory tract infections, with the most recent occurring four weeks prior, when her husband was diagnosed with influenza type A. Notably, the patient had not been vaccinated against influenza.

On admission, the patient was in a good general condition, and foetal monitoring via cardiotocography was normal. She underwent a caesarean section without immediate complications, delivering two male infants in a stable overall condition. Postoperatively, at night, the patient experienced a sudden loss of consciousness. Upon examination, her BP was at 157/90 mmHg, her heart rate (HR) was 100 bpm, and she exhibited cyanosis of the skin. After elevating her lower limbs, the patient regained consciousness within 5–7 min but had no memory of the event, only recalling dyspnoea. An initial electrocardiography (ECG) showed supraventricular regular rhythm with isolated premature ventricular contractions. A blood test revealed an increase in leukocytes (17.1 × 10^9^/L) with the dominance of neutrophils, as well as higher levels of procalcitonin (PCT), reaching 11.96 ng/mL (N < 0.5), suggesting a possible inflammatory process. Moreover, cardiac biomarkers were also significantly elevated as troponin T was 0.100 ng/mL (N < 0.014). Subsequent ECGs revealed multiple premature ventricular contractions and episodes of non-sustained supraventricular tachycardia, raising concerns for significant cardiac involvement. Echocardiography showed a left ventricular ejection fraction (LVEF) of 48–50%, with akinesis of the mid-segment of inferolateral wall, the inferior ventricular septum, as well as akinesis with features of fibrosis of the inferior wall, along with hypokinesis of the apical segment. Moreover, an enlarged left ventricular chamber was also noted (Left Ventricular End-Diastolic Dimension (LVEED) 55–56 mm), with a wall thickness within the normal range and with no significant valvular abnormalities. Pathological pericardial effusion was not observed. These findings raised concerns for PPCM. Additionally, blood and urine cultures were negative, and chest X-ray, abdominal ultrasound, brain computed tomography (CT), and Doppler of the carotid arteries and the lower limb veins were normal.

Over three days, the patient’s PCT levels decreased to 1.31 ng/mL, troponin T levels to 0.027 ng/mL, and NT-proBNP levels from 4552 to 1320 pg/mL. The patient was discharged from ICU after four days with a diagnosis of left ventricular dysfunction, possibly due to post-inflammatory changes or PPCM. Her discharge medications included metoprolol 25 mg due to persistent episodes of ventricular tachycardia, and magnesium with vitamin B6 to avoid its deficiency. ACE inhibitors as well as diuretics were not administered. As she chose to continue breastfeeding, in accordance with current recommendations, the use of bromocriptine therapy was contraindicated due to its prolactin-inhibiting effect. She was scheduled for follow-up investigations, including cardiac magnetic resonance (CMR), echocardiography, and Holter ECG monitoring.

**Figure 1:**
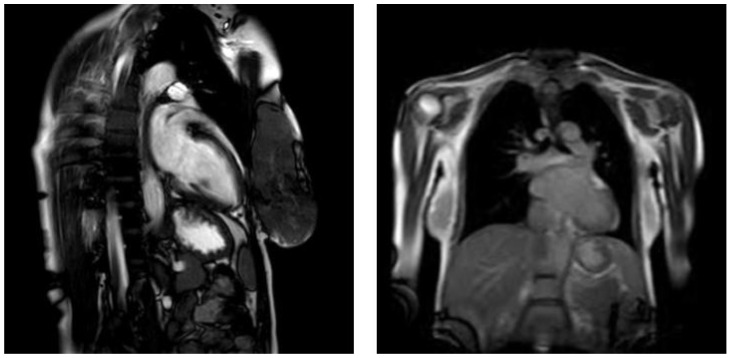
Cardiac MRI of patient reported the enlargement of left ventricle with hypokinesis of the mid-inferior and inferolateral segments one month after birth. LVEF was estimated to be 65%.

Cardiac MRI performed a month after childbirth confirmed the presence of an enlarged left ventricle with hypokinesis of the mid-inferior and inferolateral segments. The LVEF was estimated as 65%, but no radiological signs of acute myocarditis, such as tissue oedema or myocardial fibrosis, were observed. These findings were consistent with the peripartum cardiomyopathy diagnosis (Figure 1).

**Figure 2:**
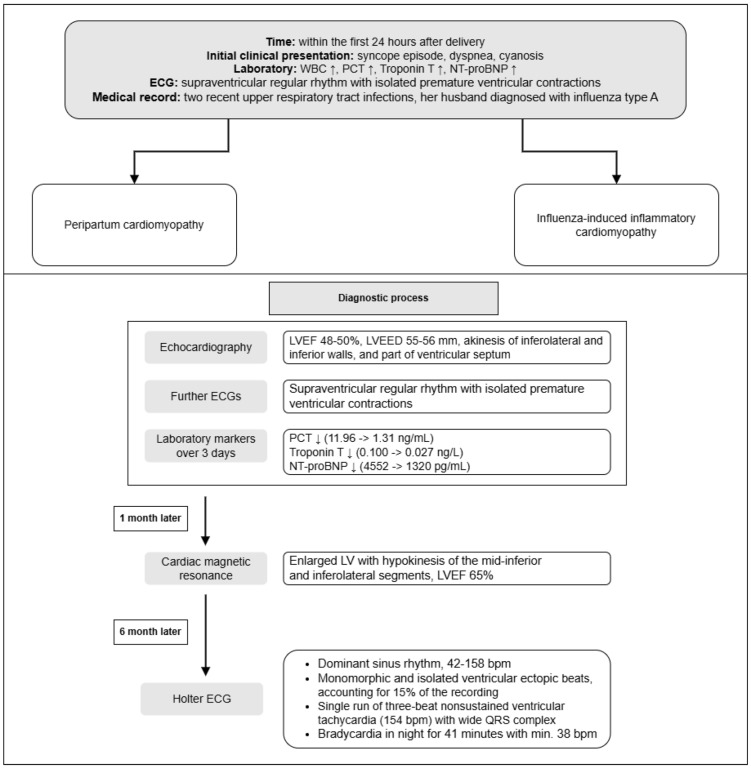
Timeline of diagnostic procedures in peripartum cardiomyopathy. (↑ increased, ↓ decreased).

A follow-up Holter ECG seven months after delivery showed dominant sinus rhythm, 42–158 bpm, with QTc interval length between 470 and 480 ms. Two episodes of sudden prolongation of the RR interval were registered during the day. Irregular rhythm was noted, partially due to frequent ventricular ectopic beats. Mostly monomorphic and isolated ventricular ectopic beats were observed, accounting for 15% of the recording. Moreover, a single run of three-beat non-sustained ventricular tachycardia (154 bpm) with a wide QRS complex was registered. Bradycardia was observed during the night and lasted 41 min with minimal 38 bpm. Therefore, the patient was referred for an ablation intervention (Figure 2).

## 5. Discussion with a Literature Review

PPCM is now widely acknowledged as a serious complication of pregnancy, presenting a life-threatening risk. It involves left ventricular dysfunction, either acute or gradually worsening, that emerges in late pregnancy (13%), during childbirth (27%), or within the postpartum period (60%), in women with no prior history of cardiac disease [1]. The prevalence is estimated to be significantly higher in the African-Americans compared to Caucasian woman [3]. Most women with PPCM present with advanced symptoms, typically classified as New York Heart Association (NYHA) class III or IV. [11]. Symptoms of heart failure, such as shortness of breath, fatigue, orthopnoea, and oedema, often mimic normal pregnancy and lead to delayed diagnosis. Physical findings may include tachycardia, elevated jugular venous pressure, and pulmonary rales, with severe cases progressing to cardiogenic shock, arrhythmias, or thromboembolic complications. Early recognition is vital to prevent complications, as some patients may progress and require advanced interventions such as mechanical circulatory support or heart transplantation [12]. The pathophysiology of PPCM is believed to involve impaired angiogenesis, influenced by vasculotoxicity from the 16 kDa prolactin fragment and the reduction in proangiogenic vascular endothelial growth factor (VEGF) expression [13]. Elevated soluble Fms-like tyrosine kinase 1 (sFlt1) levels, which antagonise VEGF and placental growth factor (PlGF), and reduced relaxin-2 levels additionally worsen angiogenesis [14,15]. What is more, increased concentrations of interleukin-6 (IL-6), tumour necrosis factor-alpha (TNF-α), and C-reactive protein (CRP) have been observed, correlating with the severity of cardiac failure [16]. Additionally, the autoimmune antibodies against cardiac sarcomeric myosin and troponin I were identified in the sera of PPCM patients, associated with a significantly lower LVEF and lower rate of recovery [17]. Alternatively, influenza-induced inflammatory cardiomyopathy results from either the direct viral invasion of myocardial tissue or exaggerated immune response triggered by influenza virus. This can lead to myocardial inflammation, mediated by elevated proinflammatory cytokines. In some instances, an autoimmune response may be triggered following the viral infection, sustaining inflammation and myocardial damage even after the virus itself has been eliminated [5]. In this case, the patient’s sudden syncopal episode after delivery, accompanied by cyanosis and elevated cardiac biomarkers, raised immediate concerns for cardiac involvement, both PPCM and ICM, given her recent history of upper respiratory infections and her husband’s confirmed influenza A infection.

The exact aetiology of PPCM still remains largely idiopathic, although several risk factors have been identified, including multiparity, multigestation, advanced maternal age, obesity or nutritional deficiencies, hypertension, pre-eclampsia, diabetes, smoking, and autoimmune diseases [3,4]. Age >30 years old, multiple pregnancy, multiparity, and infection during pregnancy were the predisposing factors for PPCM of the described patient. Moreover, genetic variants, particularly mutations in the sarcomeric gene titin (TTN), which are found in approximately 10% of women with PPCM, are reported to increase the risk. Notably, patients with TTN truncating variants (TTNtvs) tend to present with a significantly lower LVEF, indicating that TTNtvs may contribute to a more aggressive disease course of PPCM compared to other causes. Alterations in genes, such as desmoplakin (DSP), filamin C (FLNC), and BAG3, also play a significant role. All of these gene variants are also known to contribute to the development of dilated cardiomyopathy, with some speculating that the physiological stresses of pregnancy may act as a trigger for the onset of the condition [10,18,19,20]. Nevertheless, genetic testing was not performed in this case.

The diagnosis of PPCM is a process of exclusion, confirmed after ruling out the other causes of heart failure in the postpartum period, including conditions such as pre-eclampsia, myocarditis, Takotsubo cardiomyopathy, pre-existing or familial cardiomyopathies, and pulmonary embolism. Echocardiography is the primary diagnostic tool for confirming cardiac dysfunction in PPCM and assessing its severity, revealing left ventricular systolic dysfunction, often accompanied by ventricular dilation. Frequently associated findings include functional mitral regurgitation and right ventricular dysfunction. In cases where echocardiography is inconclusive, CMR can provide further details [21]. ECG findings are nonspecific but may show sinus tachycardia, ventricular hypertrophy, ST-segment deviation, or left bundle branch block [22,23]. Cardiac biomarkers like BNP, NT-proBNP, and troponins are often elevated, indicating myocardial stress or injury. Blood tests, including complete blood count and assessments of kidney, liver, and thyroid function, further aid in ruling out other systemic conditions. The patient’s echocardiography, showing reduced LVEF, akinesis, and LV dilation alterations in ECG, only confirmed myocardial dysfunction [23]. The initial laboratory findings, including elevated PCT and leucocytes levels, suggested inflammatory process involvement, though blood and urine cultures, and other imaging did not confirm an infectious source. These findings, with the lack of preexisting heart abnormalities and the normal imaging results from other systems, supported the diagnosis of PPCM. In contrast, the ICM “gold standard” diagnosis remains endomyocardial biopsy, and it may present direct evidence of myocardial inflammation through immunohistochemical analysis and the detection of viral genomes using quantitative PCR. Due to ongoing infection, oedema, hyperaemia, capillary leak, necrosis, and fibrosis are observed in CMR imagining [24,25,26]. However, when MRI findings present no definitive signs of myocarditis, PPCM remains the most likely diagnosis, as in our case. The CMR performed one month postpartum showed the absence of radiological signs of myocarditis, supporting the diagnosis of PPCM over influenza-related ICM [27]. This case supports vaccination against influenza as an essential preventive measure to reduce the risk of influenza-related heart complications, including myocarditis and subsequent cardiomyopathy [28,29] (Table 1).

Mild cases of heart failure can be managed with standard heart failure medications in general wards or even on an outpatient basis, provided that regular follow-up is guaranteed. If the patient demonstrates acute symptoms, they should be transferred to specialised centre. The treatment of acute or subacute heart failure in PPCM focuses on stabilizing the patient while avoiding medications harmful to the foetus, such as ACE inhibitors, ARBs, and MRAs. If haemodynamic instability is severe, inotropics and vasopressors are recommended, as well as mechanical circulatory support in cardiogenic shock. Loop diuretics are used to manage pulmonary congestion, with caution to avoid reducing placental blood flow. Hydralazine and nitrates can be used safely in pregnancy for patients with hypertension or severe LV dysfunction, and beta-blockers are introduced gradually [9,10]. Bromocriptine, a prolactin inhibitor, may aid in LV recovery, especially in severe cases. However, a significant limitation of bromocriptine therapy is the requirement to discontinue breastfeeding, as the drug suppresses lactation. This may affect maternal–infant bonding and pose nutritional challenges. Nevertheless, it is important to note that many women with PPCM may already be unable to breastfeed due to their clinical condition and the severity of heart failure symptoms [30,31]. Anticoagulation therapy in the prophylactic doses is also recommended, especially when thromboembolism is suspected and paired with bromocriptine therapy, considering the increased risk of those events in PPCM patients [32]. Long-term heart failure therapy should continue for at least six months after LV function recovery, followed by gradual tapering to avoid relapse. In cases of severe LV dysfunction persisting 6–12 months after initial presentation despite optimal medical therapy, the implantation of implantable cardioverter-defibrillator (ICD) or cardiac resynchronization therapy (CRT) is recommended. If arrhythmias are present, ablation intervention can be performed. For patients who do not recover after 6–12 months or when mechanical circulatory support is not feasible or appropriate, cardiac transplantation may be considered [9,10]. However, it is important to note that patients with PPCM tend to have higher rates of graft failure and mortality following heart transplantation [33].

While PPCM is managed with standard heart failure therapies, influenza-induced myocarditis may require additional antiviral or anti-inflammatory treatments [3,5]. As our patient was not suspected of ICM based on performed diagnostic process, she was treated as PPCM guidelines stated at the hospital. Despite the initial improvement, the patient continued to experience significant symptomatic arrhythmias, as revealed by the follow-up ECGs. Their persistence necessitated further intervention, including the referral for an ablation procedure and beta-blocker treatment. This highlights the importance of ongoing monitoring, not only during the initial postpartum period but extending up to five years, as PPCM is associated with risk of relapse, especially in future pregnancies [34]. Studies suggest that up to 20–33% of women who have had PPCM may experience a recurrence in subsequent pregnancies, making close monitoring and pre-conception counselling crucial [35,36,37]. The 6-month recovery rate varies depending on racial background and geographic region, ranging between 44 and 63% in Europe and the United States, and 21–36% in Africa and other low-income countries. The mortality differs between regions and economic status, being estimated as 2.5% in 1 month and 6% in 6 months [10].

## 6. Conclusions

In conclusion, this case underscores the diagnostic and therapeutic challenges in managing postpartum cardiomyopathy. The overlapping clinical features and the complex interplay of risk factors necessitate a thorough diagnostic approach, including the use of CMR, to accurately differentiate between PPCM and influenza-induced ICM. The persistence of symptomatic arrhythmias despite initial improvement highlights the need for ongoing monitoring, due to the potential relapse. Pre-conception counselling and careful planning are critical for women with a history of PPCM, as there is a significant risk of recurrence in future pregnancies. Furthermore, preventive measures such as influenza vaccination during pregnancy play a crucial role in reducing the risk of influenza-related cardiac complications, not only protecting the patient and her baby but also minimizing the risk of exposure among her close contacts.

## Figures and Tables

**Table 1:** Comparison of peripartum cardiomyopathy and influenza-induced inflammatory cardiomyopathy.

Feature	Peripartum Cardiomyopathy	Influenza-Induced Inflammatory Cardiomyopathy
Typical onset	Last month of pregnancy or within 6 months postpartum	During as well as shortly post-viral infection
Aetiology	Idiopathic in most cases, genetics 10%	Influenza infection: Direct cardiotoxicity Cytokine-mediated cardiotoxicity Autoimmune response
Risk factors	Multiparity, multiple pregnancy, advanced maternal age, obesity or nutritional deficiencies, hypertension, pre-eclampsia, diabetes, cigarette smoking, autoimmune diseases	Immunosuppression, lack of influenza vaccination
Symptoms	Fatigue, dyspnea, syncope, oedema	Similar to PPCM, however often accompanied by viral infection symptoms, like fever, myalgia, cough, sore throat
Cardiac magnetic resonance	Reduced LVEF, LV dilatation, LV hypertrophy, often oedema	Reduced LVEF, LV dilatation, oedema, hyperaemia, capillary leak, necrosis, and fibrosis
Definite diagnostic tools	Diagnosis of exclusion	Endomyocardial biopsy “golden standard”
Treatment	Standard HF therapy, bromocriptine (postpartum)	Standard HF therapy + antiviral therapy, immunosuppression
